# Conductive Polyisocyanide Hydrogels Inhibit Fibrosis and Promote Myogenesis

**DOI:** 10.1021/acsabm.4c00210

**Published:** 2024-04-09

**Authors:** Jyoti Kumari, Odile Paul, Lisa Verdellen, Bela Berking, Wen Chen, Lotte Gerrits, Jelle Postma, Frank A. D. T. G. Wagener, Paul H. J. Kouwer

**Affiliations:** † Institute for Molecules and Materials, Radboud University, Heyendaalseweg 135, 6525 AJ Nijmegen, The Netherlands; ‡ Department of Dentistry—Orthodontics and Craniofacial Biology, Radboud University Medical Centre, 6525 EX Nijmegen, The Netherlands; § Department of General Instrumentation, Radboud University, Heyendaalseweg 135, 6525 AJ Nijmegen, The Netherlands

**Keywords:** synthetic hydrogel, polyisocyanides, poly(aniline-*co*-*N*-(4-sulfophenyl)aniline), antifibrotic, myogenesis

## Abstract

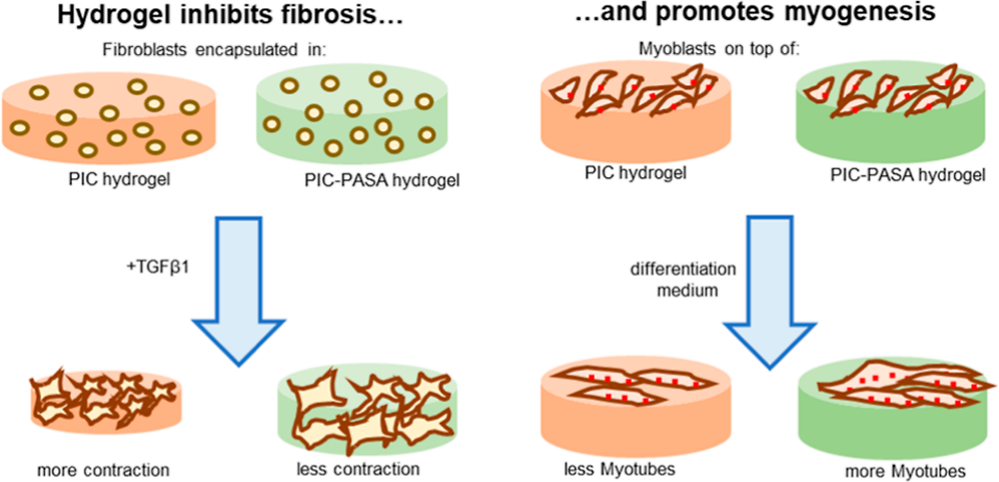

Reliable in vitro models closely resembling native tissue are urgently needed for disease modeling and drug screening applications. Recently, conductive biomaterials have received increasing attention in the development of in vitro models as they permit exogenous electrical signals to guide cells toward a desired cellular response. Interestingly, they have demonstrated that they promote cellular proliferation and adhesion even without external electrical stimulation. This paper describes the development of a conductive, fully synthetic hydrogel based on hybrids of the peptide-modified polyisocyanide (PIC-RGD) and the relatively conductive poly(aniline-*co*-*N*-(4-sulfophenyl)aniline) (PASA) and its suitability as the in vitro matrix. We demonstrate that incorporating PASA enhances the PIC-RGD hydrogel’s electroactive nature without significantly altering the fibrous architecture and nonlinear mechanics of the PIC-RGD network. The biocompatibility of our model was assessed through phenotyping cultured human foreskin fibroblasts (HFF) and murine C2C12 myoblasts. Immunofluorescence analysis revealed that PIC–PASA hydrogels inhibit the fibrotic behavior of HFFs while promoting myogenesis in C2C12 cells without electrical stimulation. The composite PIC–PASA hydrogel can actively change the cell fate of different cell types, providing an attractive tool to improve skin and muscle repair.

## Introduction

Electrical stimulation (ES) regulates some of the most fundamental cell behaviors in the human body. At the single-cell level, segregation of charges achieved by ion fluxes through ion channels and transporters generates a transmembrane potential in excitable and nonexcitable cells.^1^ Recently, surprising specificity has been uncovered in the relationship between changes in transmembrane potential levels and the alteration of cell function. Experimental evidence shows how cellular exposure to exogenous electrical signals leads to the activation of second-messenger cascades that ultimately drive transcriptional responses involved in cellular proliferation, migration, differentiation, and dedifferentiation.^2^ From this perspective, ES functions as a tool to guide cells toward a desired cellular response and has been commonly applied in tissue engineering applications, where functional biological substitutes are designed by combining cells, scaffolds, and stimulating factors to restore, maintain, or improve damaged tissues.^3^

In recent years, conducting polymers (CPs) have been frequently applied to develop electroactive culture systems. CPs such as polyaniline (PANI), polypyrrole, polythiophene, and their derivatives are attractive materials due to their high stability, tunable electrical properties, and easy synthesis.^4^ Surprisingly, even without external electric fields, CPs can capture and disseminate endogenous (bio)electrical signals that promote cellular activities, including proliferation and differentiation.^5−7^ Despite their ability to regulate gene expression and cell behavior, CPs often fail to provide an optimal surface for cellular adhesion due to their strong tendency to aggregate and their brittle nature, which strongly restricts their biomedical applications.^8^ To overcome this limitation, CPs have been incorporated into hydrogel scaffolds, generating a class of materials that is often referred to as conductive hydrogels, which has generated a range of applications in implantable devices, bioelectronics, and tissue engineering,^9−11^ up to the stage of preclinical testing. While many promising results have been obtained, including stem cells,^12^ chondrocytes, and bone cells,^13^ among others, any of these gels will not be very suitable for 3D cell culture experiments as they poorly recapitulate the native cellular microenvironment.

We follow a minimalistic approach to a conductive biomimetic hydrogel based on a well-defined synthetic hydrogel that closely resembles the architecture and mechanical properties of biological materials, together with a conductive component. The latter is supplemented at a low concentration to maintain the biomimetic character of the gel. We use hydrogels based on polyisocyanides^14^ (PICs), which form a relatively new class of tunable soft materials.^15^ By tailoring the molecular weight and polymer concentration, the mechanical properties and network architecture can be controlled for native tissue.^14,16^ As a result, the material has been successfully applied as a matrix material for 3D in vitro studies to study single cell^17,18^ and organoid behavior.^19,20^ Additional in vivo experiments indicate that PIC hydrogels are nontoxic and biocompatible.^21,22^ In these applications, PIC is biofunctionalized with the commonly used cell-adhesive peptide Gly-Arg-Gly-Asp-Ser (RGD) that allows cell–matrix interactions via integrin binding, but other biological cues can be inserted similarly.^23^ These and many other studies demonstrate the capacity of PIC gels to combine a high degree of tunability and similar mechanical properties as native tissue, which makes them particularly suitable for in vitro studies and different tissue engineering purposes.

PIC hydrogels are composed of neutral polymers and show poor conductivity. To introduce conductive properties, we prepared a hybrid gel with the CP, poly(aniline-*co*-*N*-(4-sulfophenyl)aniline) (PASA). PASA is a modified version of PANI,^24^ which has been utilized previously to generate conductive in vitro models that promote cellular activities without additional ES. For example, Srisuk and co-workers applied PANI to gellan gum spongy-like hydrogels, showing that PANI was biocompatible with L929 fibroblasts and the myoblast cell line C2C12 and promoted proliferation and myogenesis of myoblasts.^25^ Later, Noh and co-workers incorporated PANI into micropatterned poly(ethylene glycol) (PEG) hydrogels through UV-induced photolithography with photomasks.^26^ The PANI/PEG surfaces were more favorable for lysine adsorption and cell adhesion, resulting in higher C2C12 cell adhesion than plain PEG hydrogels. In yet another study, researchers developed a PANI-containing poly(vinyl alcohol) hybrid hydrogel that significantly enhanced fibroblast proliferation and adhesion in vitro.^27^ While PANI is considered a promising CP due to its tuneability, ease of synthesis, low costs, and positive effects on cellular function,^4^ its poor water solubility hinders the formation of homogeneous solutions. By incorporating phenyl-4-sulfonate groups within PANI, better processable CPs are obtained. Zhang and Guo finely dispersed PASA throughout a silk-fibroin scaffold, creating a suitable microenvironment that enhanced the myogenic differentiation of C2C12s with great potential for skeletal muscle regeneration.^28^

In this work, we combine the tailorability of the synthetic PIC-RGD gel with the conductive properties of PASA to form a fully synthetic conductive matrix. During our study, we analyzed key physical matrix properties, including stiffness, electrical resistance, and PASA entrapment inside the hydrogel. As examples of the potential of PIC–PASA hydrogels as in vitro matrix, we evaluated the proliferation and differentiation of human foreskin fibroblasts (HFF) and C2C12 myoblasts. We hypothesize that the enhanced electroactive nature of the PIC–PASA hydrogel actively changes the cell fate and promotes viability, growth, and differentiation.

## Materials and Methods

### Materials

Aniline, ammonium persulfate (APS), sodium diphenylamine-4-sulfonate, hydrogen chloride (HCl), Dulbecco’s modified Eagle’s medium (DMEM), fetal bovine serum (FBS), trypsin-ethylenediaminetetraacetic acid (EDTA), CCK-8, Triton X-100, transforming growth factor beta 1 (TGFβ1), 4′,6-diamidino-2-phenylindole (DAPI), primary antibodies against human alpha-smooth muscle actin (α-SMA), and myosin heavy chain (MyHC) were obtained from Sigma-Aldrich (St. Louis, MO, USA). Phalloidin Alexa Fluor 568, Calcein-AM, and TOTO-3 were purchased from Invitrogen (Thermo Fisher Scientific, United Kingdom). CNA35-OG488 was obtained from the Department of Biomedical Engineering (TU/e Eindhoven, The Netherlands), and Alexa Fluor 488 labeled goat antimouse secondary antibody was purchased from Molecular Probes Life Technologies.

### Synthesis of PIC-RGD

PIC polymers were synthesized following previously reported procedures.^29,30^ Briefly, PIC was prepared through copolymerization of tri(ethylene glycol)-grafted isocyano-(d)-alanyl-(l)-alanine monomer and azide-appended monomer (3.3 mol %) in the presence of a Ni(ClO_4_)_2_·6H_2_O catalyst (monomer catalyst ratio of 1000:1). The reaction was stirred in toluene overnight at room temperature (RT). The completion of the reaction was monitored by Fourier-transform infrared spectroscopy (FTIR) by monitoring the disappearance of isocyanide absorption at 2140 cm^–1^. The reaction mixture was precipitated in diisopropyl ether under vigorous stirring and collected by centrifugation. Next, the precipitate was redissolved in dichloromethane and precipitated in diisopropyl ether two more times.

PIC was biofunctionalized following our earlier published protocol.^30,31^ In short, the integrin-binding GRGDS peptide (H-Gly-Arg-Gly-Asp-Ser-OH, Bachem, Germany, 1.4 mg) was dissolved in borate buffer (0.25 mL) and reacted with DBCO-PEG4-NHS (Bioconjugate Technologies, Scottsdale, US, 2.1 mg) dissolved in DMSO (0.3 mL). The mixture was reacted at room temperature for 4 h, and full conversion was confirmed by mass spectrometry. Next, the DBCO-peptide conjugate was added to the azide-decorated PIC dissolved in acetonitrile (2.5 mg/mL) (DBCO/azide ratio of 1:1). The reaction was stirred at room temperature for 24 h. Afterward, the GRGDS-conjugated polymer (PIC-RGD) was precipitated in diisopropyl ether and collected by centrifugation. PIC-RGD pellets were air-dried, sterilized (ultraviolet (UV) light, 254 nm, 10 min), and dissolved in sterile PBS (8 mg/mL) at 4 °C overnight. Dissolved PIC-RGD was aliquoted and stored at −20 °C until further use for cell culture experiments.

### Synthesis of PASA

PASA was synthesized, as described earlier, and we followed the same procedure.^24^ In short, sodium diphenylamine-4-sulfonate (0.8831 g, 4.5 mmol) and freshly distilled aniline (0.143 g, 1.5 mmol) were dissolved in aqueous HCl (1.2 M, 15 mL). Subsequently, APS (2.052 g, 9 mmol) was dissolved in aqueous HCl (1.2 M, 20 mL) and added dropwise to the mixed solution while stirring. The mixture was stirred for 22 h at room temperature and subsequently centrifuged (20 min, 5000 rpm). The resulting precipitate was washed with 1.2 M HCl and centrifuged five times for 10 min. Lastly, the obtained dark green precipitate was dried under a vacuum for 20 h at RT. Synthesized PASA was sterilized using UV light at 254 nm (10 min) and dissolved in sterile PBS to obtain a final concentration of 1 mg/mL. Dissolving PASA in PBS was facilitated by sonicating the mixture for 20 min at 4 °C. The degree of oxidation and doping of PASA, which is crucial for its conductive state, was analyzed with UV–vis. Other common characterizations to measure, for instance, molecular weight and copolymer composition, were not pursued because the data provided in the original manuscript was difficult to interpret.

### Preparation of PIC–PASA Hydrogels

PIC–PASA hydrogels were prepared by combining the PIC-RGD stock solution (8 mg/mL) with the PASA stock solution (1 mg/mL) to obtain scaffolds with 2 mg/mL PIC and varying concentrations of PASA (0, 0.125, 0.25, 0.5, and 1 mg/mL). The obtained scaffolds were coded as PIC–PASA 0 (i.e., PIC without PASA), PIC–PASA 0.125, PIC–PASA 0.25, PIC–PASA 0.5, and PIC–PASA 1, respectively.

### Degradation of PIC

PIC was labeled with Cyanine 7 (Cy7) through the addition of dibenzocyclooctyne (DBCO)-conjugated Cy7 (Click Chemistry Tools) in a 1:500 dye/monomer ratio, followed by incubation at 4 °C for 24 h. The resulting PIC-Cy7 was then seeded into a 96-well plate (100 μL, 2 mg/mL, Falcon, 96-well clear flat bottom) and gelled for 1 h at 37 °C, followed by the addition of PBS (200 μL). On days 0, 1, 3, and 7, supernatants were taken (100 μL) and replaced with fresh PBS (100 μL). The fluorescence of the collected supernatant (100 μL) was measured on a Tecan Spark M10 plate reader (λ_ex_ = 735 nm, λ_em_ = 780 nm). A positive control containing DBCO-Cy7 (0.003 mM, similar to the PIC-Cy7 concentration in the hydrogel) was included in the study.

### Mechanical Analysis

Rheological measurements were performed on a stress-controlled rheometer (Discovery HR-1 or HR-2, TA Instruments) using a 20 mm steel parallel plate geometry with a gap of 500 μm. Different PIC–PASA 0, 0.125, and 0.25 mg/mL hydrogels were loaded as a cold (5 °C) solution on the precooled (5 °C) bottom plate. The storage modulus (*G*′) was measured in oscillatory deformation (amplitude γ = 0.04, frequency ω = 1.0 Hz) on a temperature ramp from *T* = 5 to 37 °C (heating rate 1 °C/min). The nonlinear regime of PIC and PIC–PASA hydrogels was evaluated by a prestress protocol, where the rheometer applies a constant prestress σ ranging from 0.5 to 150 Pa with a small superposed oscillatory stress δσ (with δσ < 0.1σ), measured at ω = 10–0.1 Hz and *T* = 37 °C. The protocol directly yields the differential modulus *K*′, defined as *K*′ = δσ/δγ, where δγ represents the oscillatory strain. In the results, *K*′ is plotted as a function of applied prestress σ at ω = 1.0 Hz. At low applied (pre)stress, i.e., in the linear viscoelastic regime, *K*′ = *G*′. Beyond a critical stress σ_c_, which defines the stress onset for nonlinearity and, consequently, the sensitivity to network deformation, a strong stiffening regime is entered, and the modulus increases exponentially with applied stress following *K*′ ∝ σ^*m*^. The stiffening index *m*, with the theoretical limit *m* ≤ 3/2, determines the intensity of the stiffening response.

### UV–Visible Spectroscopy of PASA

UV–vis spectroscopy was used to determine the leakage of PASA from the hybrid gel. PIC–PASA 0.25 mg/mL hydrogels were synthesized, as described in the methodology section, and subsequently heated to 37 °C. After the gel formation, at each time point, 100 μL of PBS was added on top of the scaffold and removed at different time points (0, 30 min, 60 min, 1 day, 5 days, and 7 days). PBS samples were transferred to a 96-well plate, and the absorption (280–800 nm) was measured using the Tecan Spark M10 plate reader. Absorption of different concentrations of PASA (0, 0.125, 0.25, and 0.5 mg/mL) in PBS was taken as a control.

### Conductivity

Conductivity measurements of different PIC–PASA hydrogels were carried out at 37 °C using a two-point probe method.^32^ The resistance of the samples was measured by inserting gold-plated electrodes in freshly prepared hydrogels (200 μL) in an 8-well microplate. The electrodes were connected to a digital multimeter. From the resistance, the conductivity of the scaffolds was calculated according to the following equation: *k* = *L*/*RA*, in which *k* is the conductivity (S/m), *L* is the length of the well (m), *R* is the resistance (Ω), and *A* is the cross-sectional area of the well plate (m^2^).^33^

### Bacterial Growth Assay

An overnight culture of *Pseudomonas aeruginosa* ATCC 10145 (LGC Standards, Germany) was diluted in Brain Heart Infusion Broth (Merck) supplemented with 0.04% tyloxapol and the corresponding PASA concentrations (0, 0.125, and 0.25 mg/mL) to an OD of 0.01 in a flat bottom 96-well plate. Planktonic growth was then monitored in a Plate Reader Spark M10 overnight at 24 °C by measuring the optical density at 600 nm every 30 min. Every condition was performed in triplicate.

### Cell Culture

All cell lines were maintained in growth medium consisting of DMEM (4.0 mM l-glutamine, 4500 mg/L glucose, and sodium pyruvate), 10% (v/v) FBS, and 1% (v/v) penicillin–streptomycin solution in a humidified cell culture incubator at 37 °C with 5% CO_2_. Upon reaching 80–90% cell confluency, cells were harvested using trypsin–EDTA. Fibroblasts derived from human foreskin (HFFs, 10^6^ cells/mL) were mixed homogeneously with different PIC–PASA 0, 0.125, and 0.25 mg/mL solutions and seeded in a microplate (10 μL, uncoated, Ibidi GmbH, Martinsried, Germany) or 96 well plate (100 μL). After seeding, the plates were put in the incubator (37 °C, 5% CO_2_) to induce gelation (30 min). Next, the encapsulated cells were supplied with prewarmed (37 °C) 50 or 200 μL of growth medium in the case of the microplate and 96 well plate, respectively. HFF cells were cultured for 6 days in the presence of TGFβ1 (10 ng/mL) to induce differentiation into myofibroblasts and analyzed on day 3 and day 6. Growth medium was refreshed on day 3. Note that for all cell handling in and on the PIC hybrids, medium changes and staining procedures were carried out on a hot plate (37 °C) inside the cell culture hood.

The C2C12 mouse myoblast cell line was purchased from ATCC (LGC Standards, Germany). Cells were seeded on top of the hybrid hydrogels. Before seeding, PIC–PASA 0, 0.125, and 0.25 mg/mL hydrogels were formed in a microplate and incubated for 30 min at 37 °C to induce gelation. After gel formation, C2C12 cells (10^6^ cells/mL) were added on top of the gels. The cells were left to attach and proliferate in the growth medium until reaching 80–90% confluency. Then, the C2C12 cultures were supplied with differentiation medium (DMEM, 4.0 mM l-glutamine, 4500 mg/L glucose, and sodium pyruvate, 10% (v/v) horse serum, and 1% (v/v) penicillin–streptomycin) to induce myotube formation. Cells were left to differentiate for 6 days, and the differentiation medium was refreshed daily.

### Live–Dead Assay

Live/dead cell assays were performed to determine cell viability after 6 days of seeding. Prewarmed Calcein-AM (2 mM; 1:1000, medium) and TOTO-3 (1 mM; 1:1000, medium) were added to the cell-seeded gels and incubated for 1 h (37 °C, 5% CO_2_). After incubation, the gels were washed with prewarmed PBS (37 °C). Samples were imaged on a SP8× AOBS-WLL confocal microscope (Leica Microsystems, Mannheim, Germany) at 37 °C using a laser of 488 and 647 nm for Calcein and TOTO-3, respectively.

### Cell Viability Assay

The viability of HFF and C2C12 cells was analyzed at days 1 and 6 in/on PIC–PASA 0, 0.125, and 0.25 mg/mL hydrogels using a Cell Counting Kit-8 (CCK-8) assay following the manufacturer’s instructions. In short, after removing the medium, 100 μL of prewarmed CCK-8 solution (1:10 dilution, basal medium) was added on top of the hydrogels. After 2 h of incubation (37 °C, 5% CO_2_), the absorbance was measured at 450 nm using the Tecan Spark M10 plate reader (Tecan Group Ltd., Männedorf, Switzerland). The day 6 absorbance was normalized to the day 1 value.

### Gel Contraction Assay

Gel contraction was analyzed by brightfield microscopy. Images of HFFs seeded in PIC–PASA 0, 0.125, and 0.25 mg/mL hydrogels were taken on day 3 and day 6 using an inverted microscope (2.5× objective). Contraction is calculated according to eq 1.
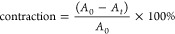
1where *A*_0_ is the area of the well, and *A*_*t*_ is the area occupied by the hydrogel at time *t*.^30^*A*_0_ and *A*_*t*_ are measured using NIH ImageJ software version 1.51.

### F-Actin Staining

Cell spreading was analyzed 6 days after seeding (for HFF cells) or 6 days after adding differentiation medium (for C2C12 cells) through visualization of the filamentous actin (F-actin) cytoskeleton networks. Before staining, cells were fixed in 4% paraformaldehyde (37 °C, 10 min) and permeabilized with 0.5% (v/v) Triton X-100 in PBS (37 °C, 20 min). Next, cells were stained for 1 h with Phalloidin Alexa Fluor 568 (400× stock solution, 1:400 dilution, PBS) and subsequently stained with DAPI (1 mg/mL, 1:100, PBS) for 30 min. Finally, hydrogels were washed with warm PBS, and samples were imaged on a SP8× AOBS-WLL confocal microscope using a 591 nm laser.

### Immunofluorescence Staining

For immunofluorescence analysis, encapsulated cells were fixed in 4% paraformaldehyde (37 °C, 10 min) and permeabilized with 0.5% (v/v) Triton X-100 in PBS (37 °C, 20 min). After washing with prewarmed PBS 0.05% (v/v) Tween-20, cells were blocked with blocking buffer (10% w/v bovine serum albumin (BSA)) for 30 min at 37 °C. The hydrogels were incubated with primary mouse antihuman αSMA (1:800 dilution, 1% BSA/PBS) or mouse monoclonal anti-MHC (1:200 dilution, 1% BSA/PBS) at 37 °C overnight. After primary antibody incubation, gels were washed in PBS 0.05% (v/v) Tween-20. Next, secondary antibody Alexa Fluor-488 labeled goat antimouse (1:200 dilution, 1% BSA PBS) was added, and the hydrogels were incubated at 37 °C for 1 h. Nuclei were stained with DAPI (1:100, PBS, 30 min) and washed with PBS before imaging on a SP8× AOBS-WLL confocal microscope.

### Total Collagen Production

Total collagen production in HFF cells was analyzed using collagen-binding adhesion protein 35-Oregon Green 488 (CNA35-OG488) following the manufacturer’s instructions. After fixation (4% paraformaldehyde, 12 min), cells were stained with CNA35-OG488 in PBS (1 μM) at 37 °C overnight. The following day, nuclei were stained with DAPI (1:100, PBS, 30 min) and subsequently washed with PBS. The hydrogels were imaged on a SP8× AOBS-WLL confocal microscope.

### Image Analysis

All acquired immunofluorescence images were processed using NIH ImageJ/FIJI software version 1.51, following previously described protocols.^34^ The size of the PASA particles inside the PIC–PASA hydrogel was analyzed using bright field images. The image was opened using the plugin “Labkit” and trained manually for background and foreground areas, after which it was converted to a probability map. After adjusting the threshold and assigning the correct pixel values, the image was used to calculate the size of the particles. The total number of particles of a given size was then calculated and converted into histograms. The results were plotted as the percentage of particles in a specific area.

The cell area, mean gray value, and integrated density were measured in randomly selected areas of interest and background regions (areas without fluorescence). The fluorescence intensity was normalized to the total number of cells in each region. The corrected total cell fluorescence (CTCF) was calculated eq 2
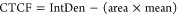
2where IntDen is the integrated density, area is the area of the selected cell, and mean is the mean fluorescence of background readings.

The fraction of viable cells was calculated from the stained images. The intensity of the green (alive) and red (dead) channels was used using eq 3(35,36)
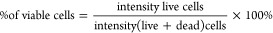
3

Myotube area, length, and width were manually quantified using NIH ImageJ/FIJI software version 1.51. The fusion index of myotubes was calculated using a macro published earlier by Hinkle et al. with some modifications^37^ using eq 4. The final macro used in this study can be found in Supporting Information.
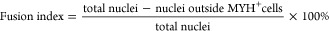
4

### Statistical Analysis

Statistically significant differences were assessed between the mean values of two groups using the Student’s *t*-test and one-way analysis of variance (ANOVA) between multiple groups. Differences were considered statistically significant at *P* < 0.05. All data in the manuscript are presented as the mean plus and minus the standard deviation. All experiments used three different samples. For image analysis, a varying number of images were taken randomly from these samples. The number of images, type of statistical test, and significance are given in the caption of the figures.

## Results and Discussion

### PIC–PASA Hydrogel Synthesis

**Figure 1:**
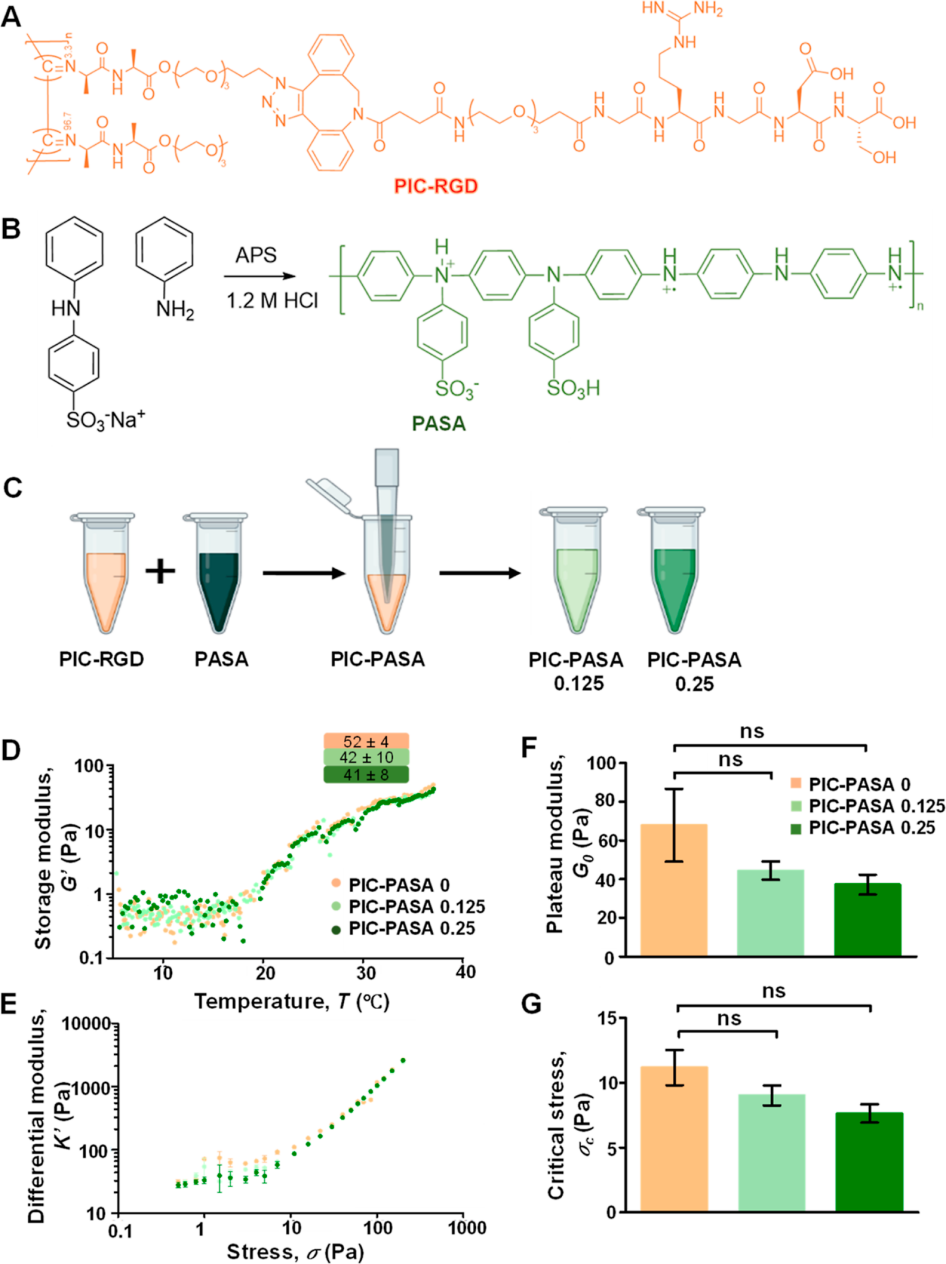
Structure and mechanical properties of PIC–PASA hydrogels. (A) Molecular structure of the PIC-RGD polymer (in orange), matching the colors in the panels. (B) Scheme for PASA polymerization. The conductive form is shown in green, matching the colors in the panels. (C) Schematic description of the preparation of the PIC–PASA hydrogels. (D) Storage modulus as a function of the temperature of PIC–PASA 0 (i.e., PIC without PASA), 0.125, and 0.25 mg/mL hydrogels. (E) Plateau modulus *G*_0_ in the linear viscoelastic regime. (F) Nonlinear mechanics: differential modulus *K*′ as a function of applied shear stress at *T* = 37 °C. (G) Critical stress σ_c_ in the nonlinear regime. For all samples: Error bars represent the standard deviation of *n* = 3 measurements. Statistical analysis with a Student’s *t*-test. *P*-values >0.05 are considered not significant (ns).

PIC decorated with azide groups (3.3 mol %) was successfully biofunctionalized with the cyclooctyne-equipped cell-adhesion peptide Gly-Arg-Gly-Asp-Ser (RGD) through the highly efficient strain-promoted azide–alkyne cycloaddition (SPAAC) reaction, following our earlier published protocol (Figure 1A).^30,38^ Upon heating, aqueous PIC-RGD solutions reversibly gel into fibrous 3D networks with micron-sized pores.^39^ The formed PIC hydrogel was checked for in vitro degradation by conjugating the polymer with Cy7 (Figure S1) and determining the leaching of polymer components in the medium. The results showed that PBS collected from the PIC-Cy7 hydrogel on day 1 and day 3, showed some fluorescence signal, which is attributed to an incomplete conjugation reaction (∼90%), but that after 7 days, no fluorescent signal could be determined in the PBS. The results suggest that the PIC hydrogel is stable and does not degrade at the time scale of the experiments considered in this work. We add that this conclusion is in line with the results obtained from PIC in vivo experiments.^21,22,40^

PASA was obtained by copolymerizing sodium diphenylamine-4-sulfonate with aniline in an aqueous HCl solution using APS as the oxidizing agent (Figure 1B). The acidic environment ensures the formation of the conductive emeraldine salt form of PASA.^24^ Solubilization of PASA in PBS by means of sonication does not yield a molecular solution but rather gives a finely dispersed dark-green suspension. The PASA dispersions settle over time in PBS. In the presence of the PIC gel, which rapidly (within seconds) forms at 37 °C, however, a fine dispersion is maintained in the pores of the gel, vide infra. The strong absorption band at 320 nm is assigned to the π–π* electronic transition of the conjugated polymer backbone.^41^

PIC–PASA hybrid hydrogels were prepared by mixing precooled PIC-RGD solution and a freshly sonicated PASA dispersion (Figure 1C). After gently pipetting the mixture up and down, gel formation was induced by heating the hybrids to 37 °C. When the temperature surpasses the PIC-RGD gelation temperature (*T*_gel_ ≈ 18 °C, Figure 1D), PIC chains quickly bundle together and form a network composed of polymer chains.^42^ This manuscript studies hybrids with the same PIC concentration (2 mg/mL) and varying PASA concentrations (0, 0.125, 0.25, 0.5, and 1 mg/mL) with the nomenclature PIC–PASA *C* mg/mL, where *C* is the PASA concentration.

### Mechanical Properties of PIC–PASA Hydrogel

The mechanical properties of the different PIC–PASA 0, 0.125, and 0.25 mg/mL hydrogels were assessed through rheology experiments. The stiffness, given by the storage modulus *G*′, was determined in the linear viscoelastic regime at 37 °C and was not affected by the different PASA concentrations in the groups at 37 °C (Figure 1D and Supporting Information Figures S3A,B). The results suggest that PIC network formation is not hindered by the (aggregated) PASA. Further analysis showed that for all scaffolds, *G*′ remains constant over the fully probed frequency regime (0.1–10 Hz) (Figure S3C), suggesting predominantly elastic behavior.

The nonlinear regimes of PIC–PASA 0, 0.125, and 0.25 mg/mL hydrogels were evaluated by a prestress protocol, where a constant prestress with a small superposed oscillatory stress is applied to the sample (Figure 1E), which directly gives the differential modulus *K*′ ≡ δσ/δγ, where δσ and δγ are the oscillatory stress and strain, respectively. Plotting the differential modulus *K*′ against prestress σ clearly shows a linear regime at low stress and a nonlinear, stiffening regime at higher stress for all samples. For different PIC–PASA 0, 0.125, and 0.25 mg/mL hydrogels, we determined the shear modulus *G*′ at low stress (Figure 1F), critical stress σ_c_ (Figure 1G), and critical strain (Figure S3D). Despite the downward trend in both the plateau modulus and the critical stress, the differences are statistically not significant, further indicating that incorporating PASA in PIC hydrogels does not significantly affect the mechanical properties of the scaffold. The results suggest that sensitivity toward stress is unaffected by PASA, and consequently, differences in cell behavior, including migration speed, should not be attributed to differences in mechanical properties.^43^ Since we know that the mechanical properties of the PIC gels are strongly dependent on the architecture of the gel,^14^ we argue that the addition of PASA does not strongly affect the fibrous structure.

### PASA Physically Captured within PIC-RGD Hydrogels

**Figure 2:**
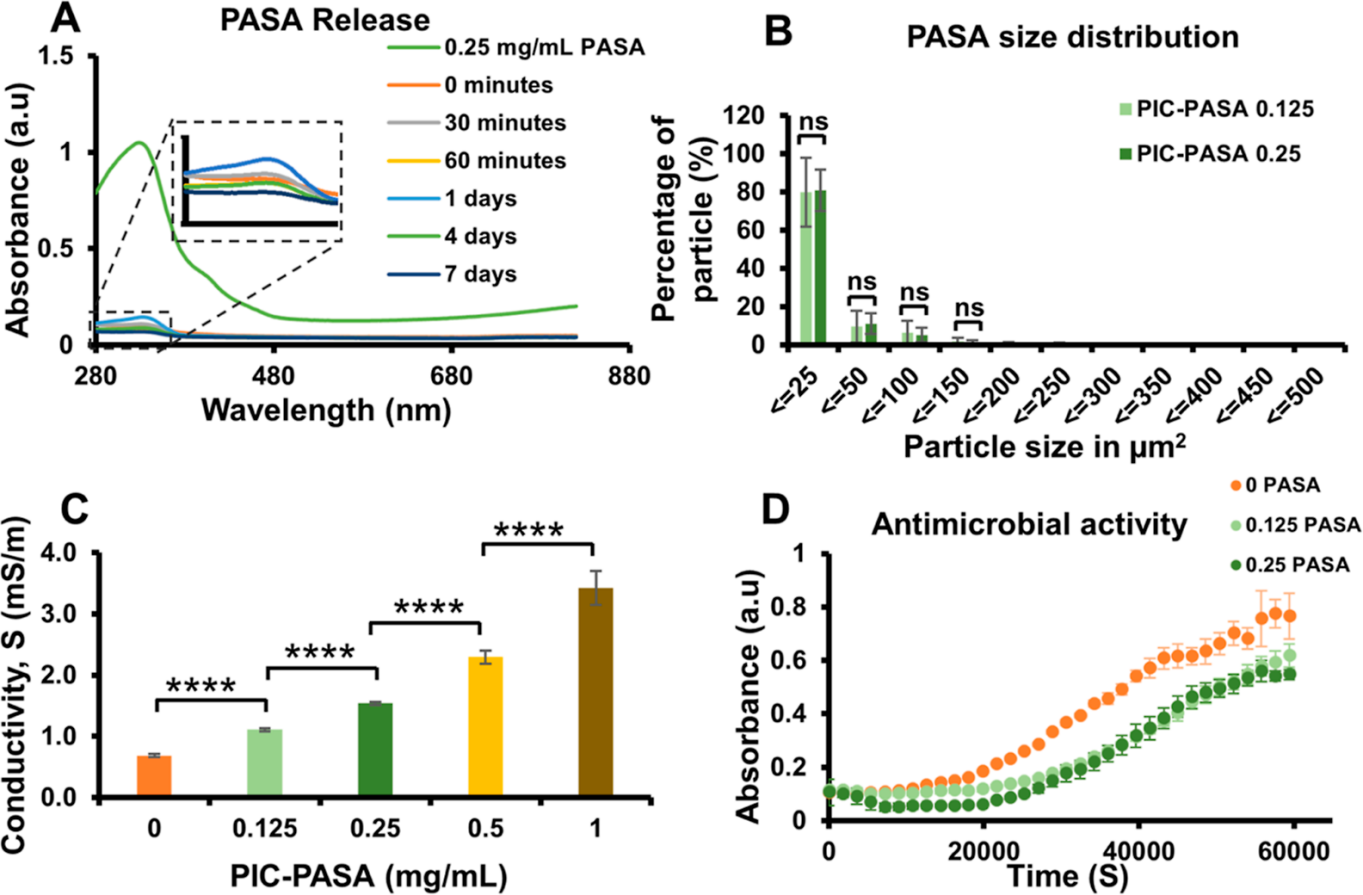
Physical properties of PIC–PASA hydrogels. (A) UV–visible spectra of PBS samples removed from PIC–PASA 0.25 mg/mL hydrogels at different time points. (B) Bright field image analysis of PASA particles inside the hydrogels. Data represented as a percentage of particle vs particle size in μm^2^. *n* = 3. (C) Conductivity of different concentrations of PIC–PASA at 37 °C; *n* = 4. (D) Optical density measured at 600 nm of *P. aeruginosa* grown with 0.125 and 0.25 mg/mL PASA and the control with 0 mg/mL PASA; *n* = 3. Statistical analysis with a Student’s *t*-test. *P*-values >0.05 are considered not significant (ns); significant differences: *, *P* ≤ 0.05; **, *P* ≤ 0.01; ****, *P* ≤ 0.0001.

The PIC–PASA hydrogels are prepared without any additional cross-linking agents, which prompted us to determine the possible leakage of captured PASA from the PIC-RGD matrix. The release was analyzed through UV–vis spectroscopy measurements of the PBS that was added on top of PIC–PASA 0.25 mg/mL hydrogel after gelation. Figure 2A confirms the absence of PASA (characteristic peak at λ = 320 nm) in the PBS samples taken at different time points, proving that PASA remains inside the matrix for at least 7 days. These results allow us to apply a straightforward approach for scaffold preparation, where well-known limitations of additional chemical cross-linking agents and side-effects such as cytotoxicity and inflammation^44^ can be avoided.

Fluorescence microscopy allows us to analyze PASA particle distribution over the PIC-RGD hydrogel as well as the distribution of particle sizes. In the image, we also stained the cell nuclei to generate the appropriate context (Figure S4 and *z*-stack videos in Supporting Information Videos S1 and S2). Qualitatively, the images show that the particles are homogeneously distributed in the gel. The particle size (distribution) was quantified by image analysis using ImageJ. We find cross sections ranging from 1 μm^2^ (lower detection limit) to 500 μm^2^. In the 3D PIC–PASA 0.25 mg/mL hydrogel, we find that the vast majority (80%) of the particles have a cross-section below 25 μm^2^ and that less than 1% of the particles forms the biggest aggregates (Figure 2B). The particle size distribution was similar for PIC–PASA at 0.125 mg/mL. To compare, the dimensions of the PASA particles are on the low-end side of commercially available PANI particles. In 2D cell culture experiments, PASA sets and form bigger aggregates that clump together at some point, which prevents further analysis (results not shown).

The uniform size distribution of PASA particles inside the hydrogel is important to maintaining its structural integrity and mechanical stability. In this study, we used sonication to dissolve and disperse PASA particles just before mixing them with PIC. Supporting Information Figures S4 and 2B illustrate particle size distribution. While most of the particles were small, we did observe some large aggregates. Improving the experimental preparation technique may make it possible to further enhance the uniformity of the size distribution of PASA particles.

### Conductivity of PIC–PASA Hydrogels

Even without ES, conductive materials have received increasing attention for in vitro applications due to their significant effects on cell fate, proliferation, and differentiation.^45,46^ We measured the conductivity of PIC–PASA 0, 0.125, 0.25, 0.5, and 1 mg/mL hydrogels at 37 °C as a function of PASA content by a two-point probe method (Figure 2C). It shows that conduction of the hybrid gels increases significantly with increasing PASA concentration *C*_PASA_, roughly scaling with *C*_PASA_^0.6^ (Figure S5), which slightly deviates from previously reported literature^47^ and Lin’s model, where the conductivity of an electrolyte scales with the square root of the electrolyte concentration. Overall, the conductivity of PIC–PASA 0.125, 0.25, 0.5, and 1 mg/mL gels is high enough to conduct microcurrents in the human body.^48,49^

### PASA Displays Antimicrobial Properties

One of the major challenges in the field of tissue engineering involves the risk of microbial infections. Traditional antibacterial agents often display disadvantages, such as toxicity for the host and only short-term antibacterial action.^50^ Consequently, the search for polymer-based scaffolds that inherently resist pathogenic infections has gained increasing attention. For example, researchers found that PANI suppresses bacterial growth by interfering with cell membrane integrity.^51^ However, the effect of PASA on microorganism viability has not been reported. We assessed the antimicrobial properties of different concentrations of PASA (0, 0.125, and 0.25) through a *P. aeruginosa* growth assay (Figure 2D). Note that we added a very low concentration of tyloxapol to prevent bacteria from forming aggregates and allow studying the direct antimicrobial effects on single bacteria instead of aggregates and biofilms. The control experiment also contains tyloxapol. We emphasize that, at this low concentration, tyloxapol has no antimicrobial effect. Analysis of the absorbance values shows a delayed increase in optical densities in the presence of PASA after 30,000 s (for 0.125 PASA: 0.53-fold, *P* = 0.0001 and for 0.25 PASA: 0.48-fold, *P* = 0.0001) and after 60,000 s (for 0.125 PASA: 0.8-fold, *P* = 0.056 and for 0.25 PASA: 0.71-fold, *P* = 0.01) compared to bacteria grown without PASA. This data indicate that the presence of PASA leads to decreased bacterial proliferation. We note that PASA did not achieve a full cytotoxic effect compared to well-known antibiotics.^52^ Yet, the observed deferred bacterial growth highlights that PASA can achieve antibacterial effects, which is highly beneficial in wound healing applications.^53^

### Biocompatibility of PIC–PASA Hydrogels

To determine the effect of PASA on mammalian cell viability, we performed a Live–Dead assay on HFF (human) and C2C12 (mouse) cells in different PIC–PASA 0, 0.125, and 0.25 mg/mL hydrogels. HFF cells were encapsulated in the hydrogel solution and supplied with TGFβ1 (10 ng/mL) to induce their differentiation into myofibroblasts.^30^ Myofibroblasts are the main extracellular matrix (ECM)-secreting cells during pathological tissue repair (and fibrosis) and are responsible for the contractility of scar tissue. In this manuscript, we consider fibroblasts cultured in the presence of TGFβ1 as myofibroblasts, which was confirmed earlier.^30^

**Figure 3:**
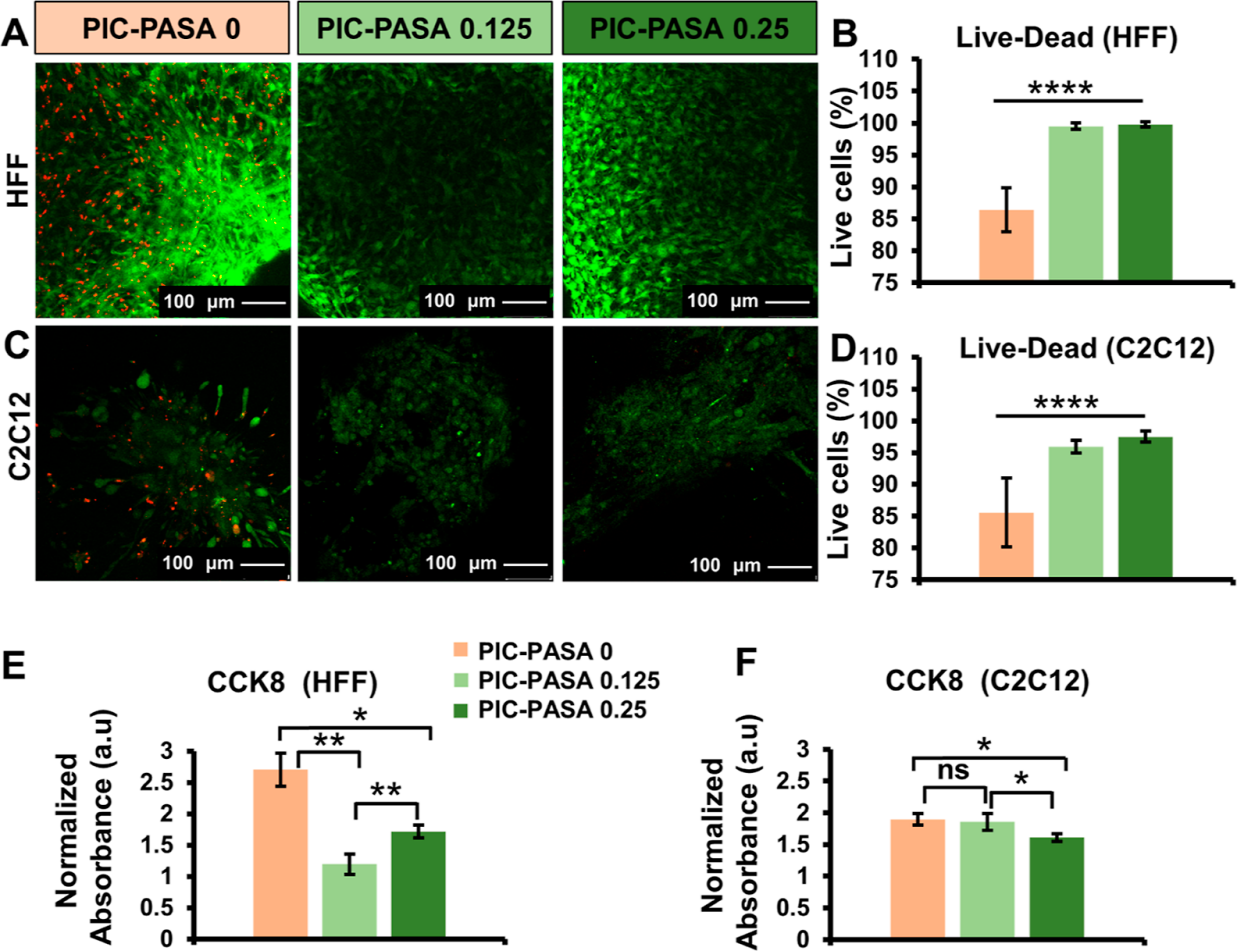
Biocompatibility study of PIC–PASA hydrogels at day 6. (A, C) Confocal images of Live–Dead assays, represented as the maximum projection of *Z*-stacks with a thickness of 100 μm. Living cells and dead cells were stained with Calcein-AM (green) and TOTO-3 (red), respectively. Scale bar = 100 μm. (B, D) Quantitative analysis of the Live–Dead confocal images. Data are given as the percentage of living cells. Panel B, *n* = 11 images; Panel D, *n* = 24 images. (E, F) CCK8 assay data as a measure for mitochondrial activity at day 6, normalized relative to day 1 value, *n* = 3. (B, D) Statistical analysis with a one-way ANOVA. (E, F) Statistical analysis with a Student’s *t*-test. *P*-values >0.05 are considered not significant (ns); significant differences: *, *P* ≤ 0.05; **, *P* ≤ 0.01; ****, *P* ≤ 0.0001.

The Live–Dead assay (Figure 3A) shows that most myofibroblasts were alive (green) after 6 days of culturing, regardless of the increase in PASA content, suggesting that PASA does not negatively affect cell viability. The biocompatibility of our hydrogels was quantified by calculating the CTCF of green (alive) and red (dead cells) channels (Figure 3B). While the cell viability is above 85% in all groups, the percentage of living cells appears to be significantly higher in PIC–PASA 0.125 mg/mL (1.15-fold) and 0.25 mg/mL (1.15-fold) hydrogels compared to PIC–PASA 0 mg/mL hydrogel. This difference may originate from the fact that the PIC–PASA 0 mg/mL hydrogels are heavily contracted as a result of myofibroblast activity and that these cells are more prone to undergo apoptosis in contracted, more densely populated tissues. No significant differences were found between PIC–PASA 0.125 mg/mL and PIC–PASA 0.25 mg/mL, indicating that cell viability was similar in both groups despite increased PASA content.

C2C12 myoblasts were also used to assess the biocompatibility of different PIC–PASA hydrogels for future skeletal muscle tissue engineering applications. Contrary to HFF cells, C2C12 cells were seeded on top of the hydrogel to help facilitate cell–cell interactions and eventually promote the formation of myotubes.^54^ In Figure 3C, the abundant green fluorescent signal reveals highly viable C2C12 cells in all groups (Figure 3C). After quantifying the Live–Dead data, however, the percentage of living C2C12 cells appeared to be higher in the presence of PASA (Figure 3D) (PIC–PASA 0.125 mg/mL = 1.12-fold, PIC–PASA 0.25 mg/mL = 1.13-fold). Tentatively, we attribute this difference to the fact that in PIC–PASA 0 mg/mL, the cellular surface area became overcrowded after seeding, which often leads to cell death as nutrients become depleted and cells start to compete for space.^55^ Although the Live/Dead data are significantly different in the presence and absence of PASA, we note that in all groups, more than 85% (or more than 95% in the presence of PASA) of all cells were viable. In summary, the Live/Dead data support our hypothesis that PIC–PASA scaffolds do not negatively affect the cell viability of C2C12 and HFF cells.

Besides viability, we also analyzed the change in mitochondrial activity through CCK8 assays between days 1 and 6 postseeding. Figure 3E,F show absorbance values for HFF and C2C12 cells seeded in different PIC–PASA hydrogels, normalized to the absorbance at day 1. For HFFs, the mitochondrial activity is higher in PIC–PASA 0 mg/mL hydrogels compared to PIC–PASA 0.125 mg/mL (2.26-fold) and 0.25 mg/mL hydrogels (1.57-fold), suggesting the cellular proliferation of myofibroblasts is significantly higher in the absence of PASA. While TGFβ1 induces myofibroblast proliferation and formation, PASA significantly inhibits these pathophysiological processes.^56^ Mitochondrial activity was further assessed in C2C12 myoblasts between days 1 and 6 postseeding. Figure 3F shows that the highest PASA concentration lowers myoblasts’ proliferation rate, as the normalized absorbance values are lower in PIC–PASA 0.25 hydrogels compared to PIC–PASA 0 and PIC–PASA 0.125 hydrogels. We believe that the enhanced conductivity of our PIC–PASA 0.25 scaffolds stimulates the transition of myoblast proliferation toward differentiation. When given proper stimuli, proliferating C2C12 cells pause in the G1 phase, permanently exiting the cell cycle and eventually starting differentiation and fusion into multinucleated myotubes.^57^ The change in cell fate due to the high concentration of PASA may explain the significantly lower proliferation rates in PIC–PASA 0.25 mg/mL.

Analysis of *z*-stacks obtained from confocal fluorescence experiments shows that PASA does not affect the cell distribution inside the hydrogel (Supporting Information Videos S1 and S2). The fluorescent images clearly show that cells grow throughout the hydrogels in the presence of PASA particles (Figure S4).

### PIC–PASA Hydrogels Show less Contraction

**Figure 4:**
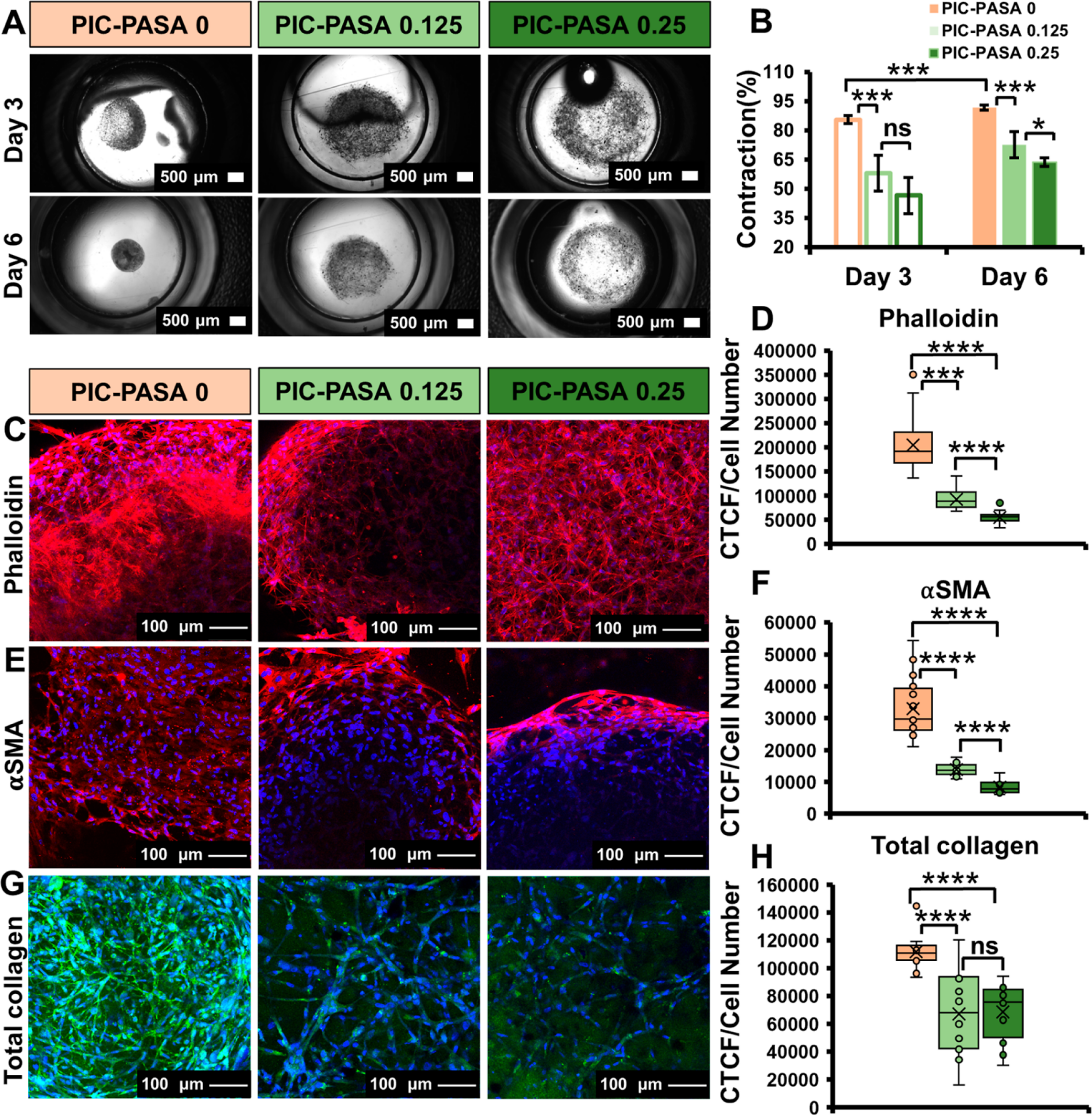
Effects of PIC–PASA hydrogel on HFF cell behavior. (A) Brightfield images showing contraction of HFFs exposed to TGFβ1 in PIC–PASA 0, 0.125, and 0.25 mg/mL hydrogels at day 3 and day 6 after seeding. (B) Quantification of the contraction in PIC–PASA 0, 0.125, and 0.25 mg/mL hydrogels at day 3 and day 6 after seeding; *n* = 3. (C, E, G) Confocal fluorescent images of F-actin stained with phalloidin Alexa Fluor 568 staining (red) (C), of αSMA immunostaining (red) (E), and of collagen stained with CNA35-OG488 (green) (G), counterstained with DAPI to visualize the nuclei (blue). The fluorescent images were captured on day 6 and represented as the maximum projection of the *z*-stacks with a thickness of 50 μm (C, G), and 100 μm (E). (D, F, H) Quantitative image analysis of the phalloidin (D), αSMA (F), and total collagen (H) confocal images at day 6 by CTCF. Intensities are normalized to the number of cells at day 6. Panel D *n* = 24 images; Panel F *n* = 16 images; Panel H *n* = 12 images. Scale bars = 100 μm. Statistical analysis with a Student’s *t*-test. *P*-values >0.05 are considered not significant (ns); significant differences: *, *P* ≤ 0.05; **, *P* ≤ 0.01; ***, *P* ≤ 0.001; and ****, *P* ≤ 0.0001.

Macroscopic hydrogel contraction was measured 3 and 6 days after HFF cell encapsulation (in the presence of TGFβ1) using brightfield imaging (Figure 4A). The gel contraction assay is based on a recently reported synthetic polymer-based 3D model, described earlier by us, where the contraction of PIC hydrogels functions as a readout for fibroblast differentiation into myofibroblasts.^30^ The results are quantified as the relative decrease in the hydrogel area compared to the hydrogel area after seeding, i.e., the area of the well. As expected, in the presence of TGFβ1, all hydrogels contracted on days 3 and 6 post HFF encapsulation (Figure 4B), suggesting that fibroblasts differentiated into contractile myofibroblasts. The hydrogel contraction was significantly lower in the presence of PASA (both PIC–PASA 0.125 and 0.25 mg/mL) compared to the control PIC gel without PASA. Furthermore, the decrease in contraction seemed to be PASA concentration-dependent, as PIC–PASA 0.25 mg/mL was less contracted than PIC–PASA 0.125 mg/mL (Day 3:0.80-fold, day 6:0.87-fold). We postulate that the loss of contractility in PIC–PASA 0.125 and 0.25 mg/mL hydrogels is caused by an inhibitory effect of PASA on fibroblast differentiation or myofibroblast functionality. Where earlier literature has shown that the presence of CPs can significantly enhance cell adhesion and proliferation of fibroblasts,^58^ the effect of CPs on the differentiation of fibroblasts into myofibroblasts has not yet been published.

### PIC–PASA Hydrogels Reduce F-Actin Stress Fiber Formation in HFFs

Besides being characterized by a well-developed contractile apparatus, myofibroblasts are also known for forming robust stress fibers by assembling actin filaments (F-actin).^59^ These prominent cytoskeletal structures contribute to migration, matrix remodeling, and transcriptional and translation regulation, demonstrating a central role in fibrosis.^60^ The formation of small contractile units, named stress fibers, has been suggested to be the most effective way to induce a high net force on a matrix.^61^ A study by Doolin and co-workers confirmed that F-actin expression is increased upon myofibroblast formation through TGFβ1 stimulation.^62^ In the PIC–PASA hybrid gels, F-actin stress fibers were visualized through staining with phalloidin Alexa Fluor 568 to obtain insight into the formation of cytoskeletal structures. The fluorescent images were quantified and scaled to the number of cells to give corrected CTCF values. At day 6 after seeding, more F-actin is observed in PIC–PASA 0 mg/mL hydrogels compared to PIC–PASA 0.125 and 0.25 mg/mL hydrogels, as CTCF values were significantly lower, 0.44-fold and 0.26-fold, respectively. Moreover, the decrease in F-actin formation seems to be concentration-dependent, as CTCF values of PIC–PASA 0.25 mg/mL were significantly lower compared to PIC–PASA 0.125 mg/mL. Quantification of the fluorescent data indicate that (myo)fibroblasts cultured in the presence of PASA express less F-actin and are, therefore, less prone to developing a cytoskeletal structure that corresponds to the phenotypic characteristics associated with fibrosis.

### PIC–PASA Hydrogel Reduces αSMA and Total Collagen Levels in Human Fibroblasts Exposed to TGFβ1

Excessive tissue contraction during fibrosis results in scarring and undesirable contractures. Myofibroblasts can pull on their surrounding tissue due to interactions between αSMA and myosin; in fact, contractile properties and αSMA expression are directly correlated.^63^ Increased expression of αSMA in response to TGFβ1 is associated with the enhanced development of stress fibers required to generate mechanical tension.^64^ To assess the effects of PASA on αSMA production in the 3D hybrid cultures, we stained myofibroblasts with primary mouse antihuman αSMA (Figure 4E) and quantified the acquired immunofluorescence images with CTCF (Figure 4F). Our results show a significant decrease in αSMA expression in PIC–PASA 0.125 and 0.25 mg/mL hydrogels (0.42-fold and 0.25-fold, respectively) compared to PIC–PASA 0 mg/mL hydrogels. The quantitative data indicate that PASA decreases αSMA levels in myofibroblasts.

During fibrosis, collagen deposition by myofibroblasts increases as the synthesis and excretion of new collagen exceeded the degradation rate.^65^ The total amount of collagen was visualized through staining with the fluorescently labeled collagen-adhesion protein CNA35-OG488 (Figure 4G). Fluorescence intensity quantification by CTCF (Figure 4H) shows significantly lower collagen levels in the PIC–PASA 0.125 and 0.25 mg/mL hydrogels compared to the PIC–PASA 0 mg/mL gel (0.60-fold and 0.61-fold, respectively). Our data show that the deposition of collagen by myofibroblasts is reduced in the presence of PASA. While further studies to fully elucidate the physiological role of PASA on myofibroblasts are still necessary, our data suggest that our PIC–PASA hydrogel inhibits the expression of myofibroblast markers and collagen production and, hence, potentially exhibits antifibrotic properties.

### PIC–PASA Hydrogel Reduces F-Actin Stress Fiber Formation in C2C12 Cells

**Figure 5:**
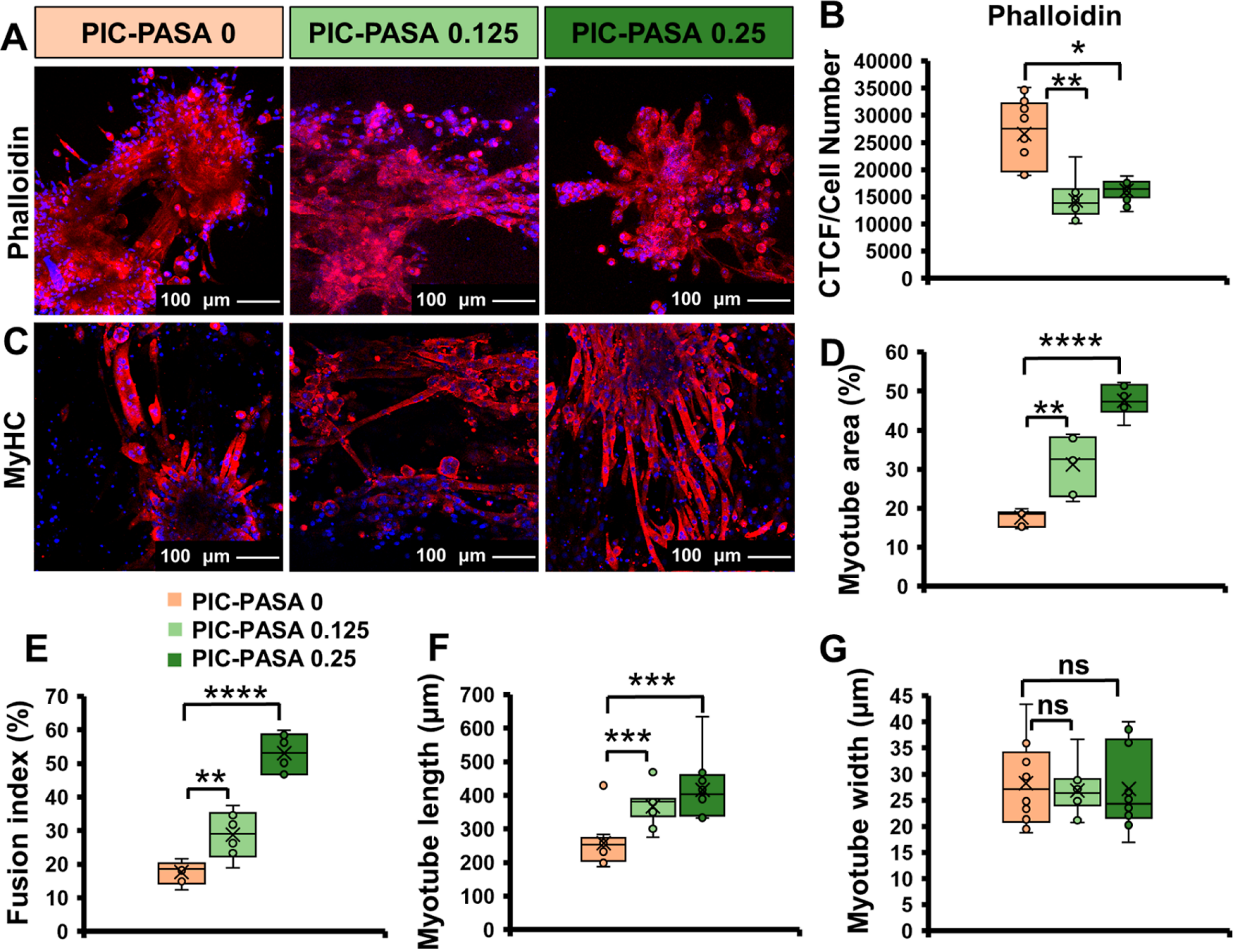
Effect of PIC–PASA hydrogel on C2C12 differentiation at day 6. (A, C) Confocal fluorescent images of F-actin stained with phalloidin Alexa Fluor 568 (red) (A), MyHC immunostaining (red) (C), counterstained with DAPI to visualize the nuclei (blue). Images in panel A represent the maximum projection of the *Z*-stacks with a thickness of 100 μm. All images in panel C were represented as a single plane. (B) Quantitative image analysis of the F-actin confocal images by CTCF; intensities are normalized to the number of cells; *n* = 12 images. (D–G) Quantitative image analysis of MyHC production: myotube area (D), fusion index (E), myotube length (F), and myotube width (G). Panels D, G *n* = 6 images; Panels E, F *n* = 10 images. Scale bar = 100 μm. Statistical analysis with a Student’s *t*-test. *P*-values >0.05 are considered not significant (ns); significant differences: *, *P* ≤ 0.05; **, *P* ≤ 0.01; ***, *P* ≤ 0.001; and ****, *P* ≤ 0.0001.

The properties of the microenvironment strongly influence myoblast cell behavior and morphology. Cells continuously probe mechanical cues and adapt accordingly by regulating the formation of actin stress fibers and tensile actomyosin.^66^ Hence, we analyzed the presence of F-actin stress fiber expression in C2C12 cells in different PIC–PASA 0, 0.125, and 0.25 mg/mL hydrogels through phalloidin staining (Figure 5A) with subsequent fluorescence intensity quantification by CTCF (Figure 5B). After 6 days of culturing, increased F-actin levels are observed in PIC–PASA 0 mg/mL hydrogels (1.8-fold and 1.6-fold, respectively) compared to PIC–PASA 0.125 and 0.25 mg/mL hydrogels.

F-actin staining did not clearly indicate the formation of tube-like structures in either of the hydrogel conditions. As phalloidin indiscriminately stains both myoblasts and myotubes, the overlapped emission does not return tube-like structures. To confirm the presence of myotubes, we performed immunostaining for a myogenic differentiation marker.

### PIC–PASA Hydrogel Promotes Myogenic Differentiation

The effect of different PASA concentrations (0, 0.125, and 0.25 mg/mL) on myogenesis was investigated. Myogenesis refers to generating muscular tissue regulated by a cascade of transcription factors. During myogenesis, proliferated myoblasts, which are embryonic precursor muscle cells, align and fuse to give rise to differentiated multinucleated myotubes.^67^ In time, the myotubes mature into skeletal muscle fibers through the expression of various transcription factors and signaling molecules.^68^ In our study, the morphology of myotubes was visualized by the myogenic differentiation marker myosin heavy chain (MyHC), the motor protein of muscle thick filaments.^69^ After 6 days in the differentiation medium, all C2C12 myoblasts on PIC–PASA hybrid gels show the formation of multinucleated myotubes (Figure 5C). Fluorescent images show how myotubes maintain an elongated morphology and contain three or more nuclei within a single membrane structure.

More insight into myotube generation was obtained by quantifying the myotube area (Figure 5D), fusion index (Figure 5E), myotube length (Figure 5F), and myotube width (Figure 5G). As shown in Figure 5D, myotubes contain a significantly larger area in PIC–PASA 0.125 and 0.25 mg/mL hydrogels than in PIC–PASA 0 mg/mL hydrogels (1.77-fold and 2.7-fold, respectively). Similarly, myotubes were significantly longer in PIC–PASA 0.125 and 0.25 mg/mL hydrogels in contrast to PIC–PASA 0 mg/mL hydrogels (1.43-fold and 1.62-fold, respectively). Since long and large myotubes indicate long-term myoblast adhesion,^70^ we suggest that cellular adhesion to PIC hydrogels is improved in the presence of PASA. The fusion index (Figure 5E) determines the percentage of nuclei within differentiated myotubes.^37^ Since this index is based on direct monitoring of myotube formation, it can be used to reliably estimate the extent of myogenesis.^71^Figure 5E shows how myotubes developed in the presence of PASA contained a significantly larger fusion index than those developed in the absence of PASA. Compared to PIC–PASA 0 mg/mL hydrogels, PIC–PASA 0.125 and 0.25 mg/mL hydrogels greatly enhanced myotube length, area, and fusion index.

As for the myoblasts, molecular mechanisms for stimulating myotube formation remain unclear. We can speculate that the conductive PIC–PASA hydrogels act as transmitters of endogenous (bio)electrical signals, simultaneously increasing the intracellular calcium level of the C2C12 cells. Porter and co-workers demonstrated that intracellular calcium levels play a crucial role in muscle cell maturation and that lowering intracellular calcium levels inhibits the differentiation of skeletal myoblasts.^72^

## Conclusions

In this study, we successfully prepared fully synthetic hydrogels from PASA and PIC to serve as stable, conductive in vitro microenvironments. The scaffolds are prepared by straightforwardly mixing aqueous solutions of the polymers and heating them to 37 °C, where the gel is formed and PASA does not leak out. Incorporating PASA introduces conductivity without altering the fibrous architecture or the linear and nonlinear mechanics of the biomimetic PIC network, which are crucial parameters in tissue engineering. Biocompatibility studies confirmed that the hybrid gels exhibit sufficient support for cell viability and proliferation. The conductivity of different PIC–PASA hydrogels increased with PASA concentration; for PIC–PASA 0.125 and 0.25 mg/mL hydrogels, conductivity values are adequate for cellular applications. Simultaneously, we observed that the PIC–PASA hydrogels exhibit antibacterial characteristics, which is particularly beneficial for wound healing applications.^73^

The presence of CPs strongly influences cell survival and behavior. We looked in more detail at fibroblasts exposed to TGFβ1 that induce differentiation into myofibroblasts and at C2C12 myoblast differentiation into myotubes. After 6 days of culturing, the vast majority of myofibroblasts and C2C12 cells remained alive in all PIC–PASA hydrogels, which is in line with previous reported data, as several researchers demonstrated the positive effects of conductive models regarding cellular adhesion and viability.^25−27,74^ The quantified data from CCK8 analysis and Live/Dead staining confirms that our conductive hydrogel provides a cytocompatibility environment for cell cultures.

In the fibroblast cultures, we found that PASA significantly inhibits contractility, one of the key players in the development of fibrosis. Furthermore, results obtained from immunofluorescence analyses showed that PASA reduced the levels of myofibroblast differentiation markers αSMA and collagen, suggesting inhibition of fibroblast differentiation into myofibroblasts despite the TGFβ1-rich environment. Quantitatively, we observed that the decrease in αSMA expression in the presence of PASA is in the same order of magnitude as what is realized with antifibrotic drugs. In a similar setup performed earlier by our group (same cells, same culture conditions), the treatment of myofibroblast with nintedanib and pirfenidone led to a similar decrease compared to nontreated myofibroblast.^30^ Overall, the results indicate that PASA likely mediates antifibrotic effects, although the underlying molecular mechanisms at the early stage of contractile or fibrotic diseases to prevent the progression of contractile lesions remain to be elucidated.

Based on earlier data, we anticipated for the C2C12 cells that PASA would promote myogenic differentiation and maturation.^25,26,74^ Indeed, compared to the nonconductive control, C2C12 cells cultured in the presence of PASA generated larger myotubes with increased fusion indices, underlining its positive effect on myogenesis. In this current study, our conductive hydrogel systems were not configured with an external electrical field. However, the system can be used to study the effect of electric fields on cell behavior. We anticipate further improvements in myogenic differentiation may occur upon stimulation with exogenous electrical fields.

In future studies, our PIC–PASA hydrogels can provide critical insight into cellular behavior in the presence of conductive polymers and subsequently function as a starting point for various tissue engineering applications, including wound repair, muscle regeneration, and antifibrotic drug testing.

## Supporting Information Available

Confocal fluorescence Z-stack videos of the PIC=PASA composite with foreskin fibroblasts (AVI)
PASA white; cell nuclei blue (AVI)
Degradation of PIC in PBS, UV-visible spectrum of PASA, mechanical properties of PIC-PASA hydrogels, PASA particles in PIC-PASA hydrogels, conductivity of PIC-PASA hydrogels, macro used to analyze fusion index (PDF)

## Supplementary Material

mt4c00210_si_001.avi

mt4c00210_si_002.avi
