# Supplementary material for: Conductive Polyisocyanide Hydrogels Inhibit Fibrosis and Promote Myogenesis
Source: ACS Appl Bio Mater. 2024 Apr 9;7(5):3258–70. doi: 10.1021/acsabm.4c00210 (PMC11110048; doi:10.1021/acsabm.4c00210)
Supplement: Supplementary file 3 — mt4c00210_si_003.pdf [file mt4c00210_si_003.pdf]

Supporting information for

Conductive Polyisocyanide Hydrogels Inhibit Fibrosis and Promote Myogenesis

Jyoti Kumari,[a,b] Odile Paul,[a] Lisa Verdellen,[a] Bela Berking,[a] Wen Chen,[a] Lotte Gerrits,[a] Jelle Postma,[c] Frank A. D. T. G. Wagener,[b,]* and Paul H. J. Kouwer[a,]*

[a] Institute for Molecules and Materials, Radboud University, Heyendaalseweg 135, 6525 AJ, Nijmegen, The Netherlands.

[b] Department of Dentistry - Orthodontics and Craniofacial Biology, Radboud University Medical Centre, 6525 EX Nijmegen, The Netherlands

[c] Department of General Instrumentation, Radboud University, Heyendaalseweg 135, 6525 AJ, Nijmegen, The Netherlands.

*Email: Frank.Wagener@radboudumc.nl; p.kouwer@science.ru.nl

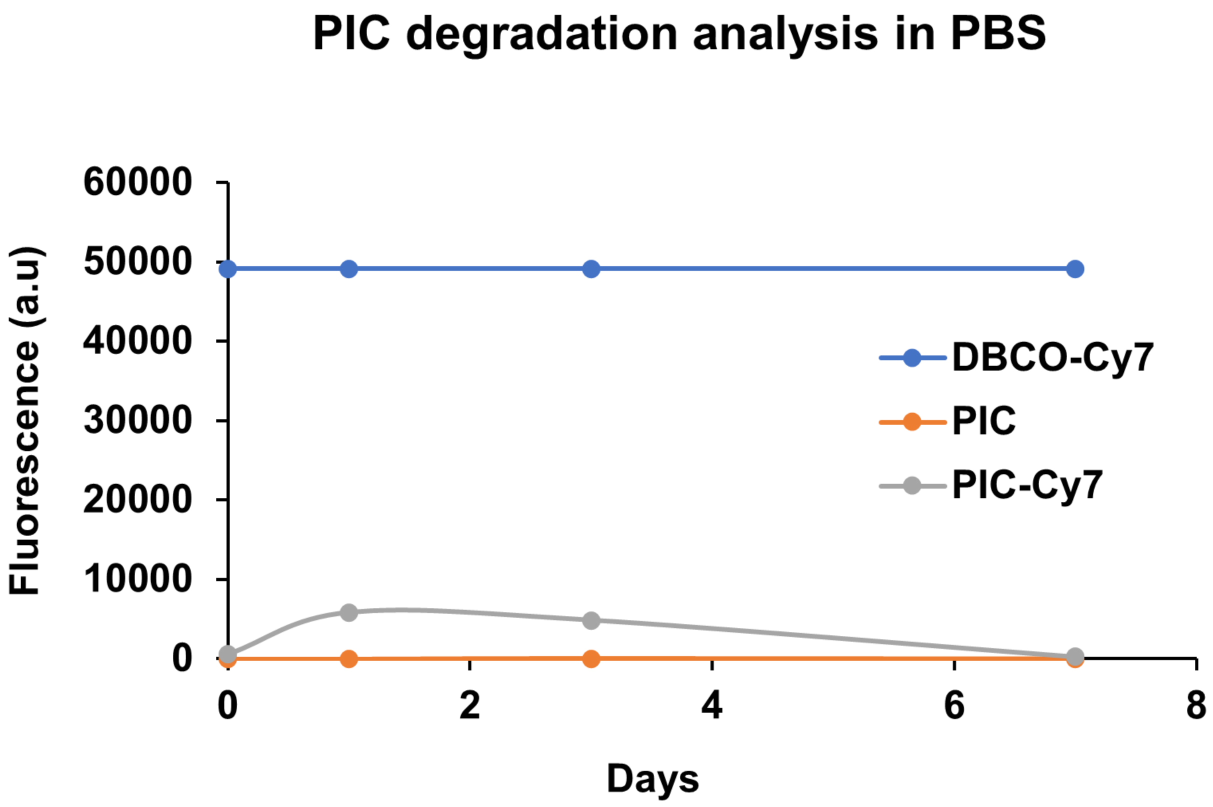

Figure S1: Degradation of PIC in PBS. Fluorescence signals of Cy-7 at different time points in case of PIC and PIC-Cy7 hydrogel; *n*=3.

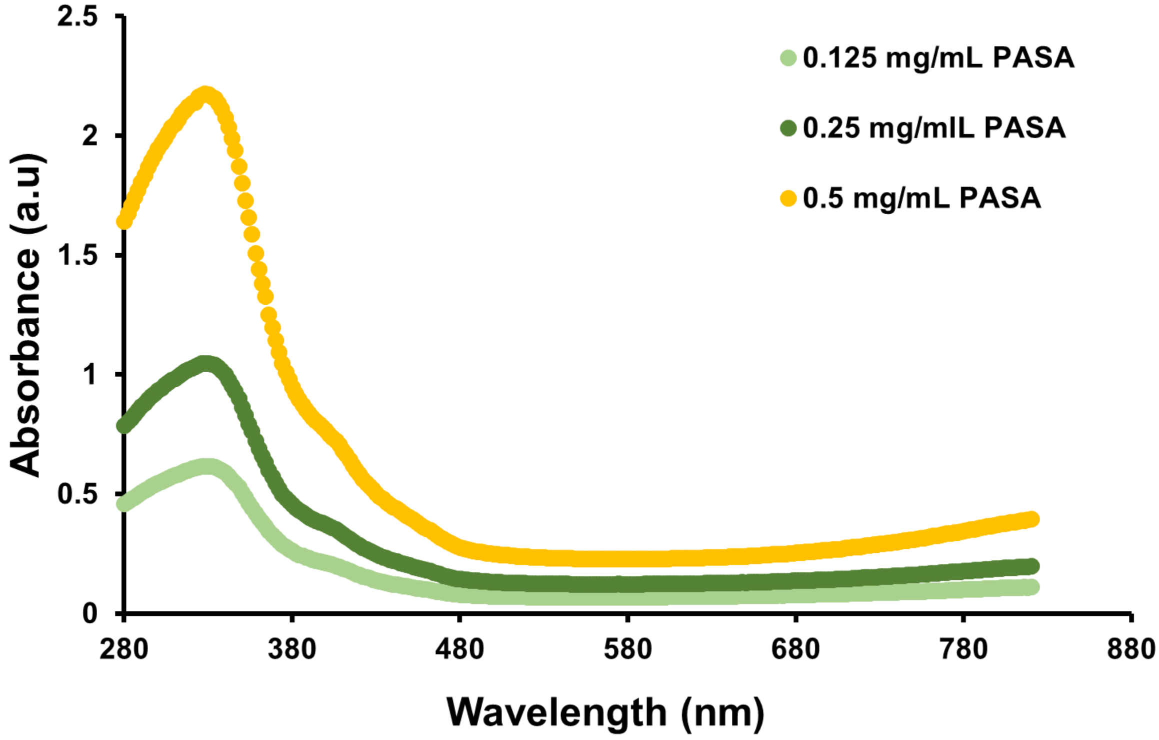

Figure S2: UV-visible spectrum of PASA. Absorption spectra (280-800 nm) of different concentrations of PASA; *n*=3.

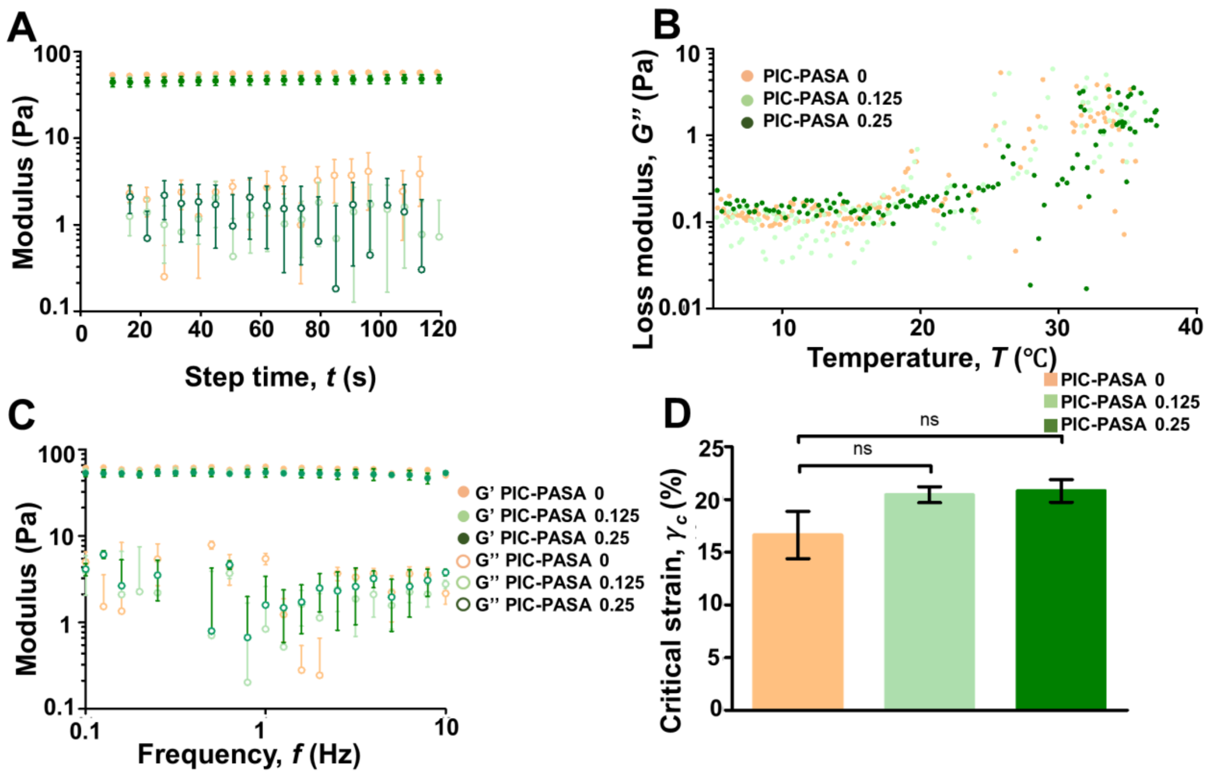

Figure S3: Mechanical properties of PIC-PASA hydrogels. A) Storage modulus G' and loss modulus G'' as a function of time of PIC-PASA 0, 0.125, and 0.25 mg/mL hydrogels at T = 37 °C. B) Loss modulus G'' as a function of temperature of PIC-PASA 0, 0.125, and 0.25 mg/mL hydrogels. C) Storage modulus G' and loss modulus G'' as a function of frequency of PIC-PASA 0, 0.125, and 0.25 mg/mL hydrogels at T = 37 °C. D) Critical strain γ_c in the nonlinear regime of PIC-PASA 0, 0.125, and 0.25 mg/mL hydrogels at T = 37 °C. Error bars represent the standard deviation of n = 3 measurements. Statistical analysis with a Student's t test. P-values > 0.05 are considered not significant (ns). For all samples; n=3.

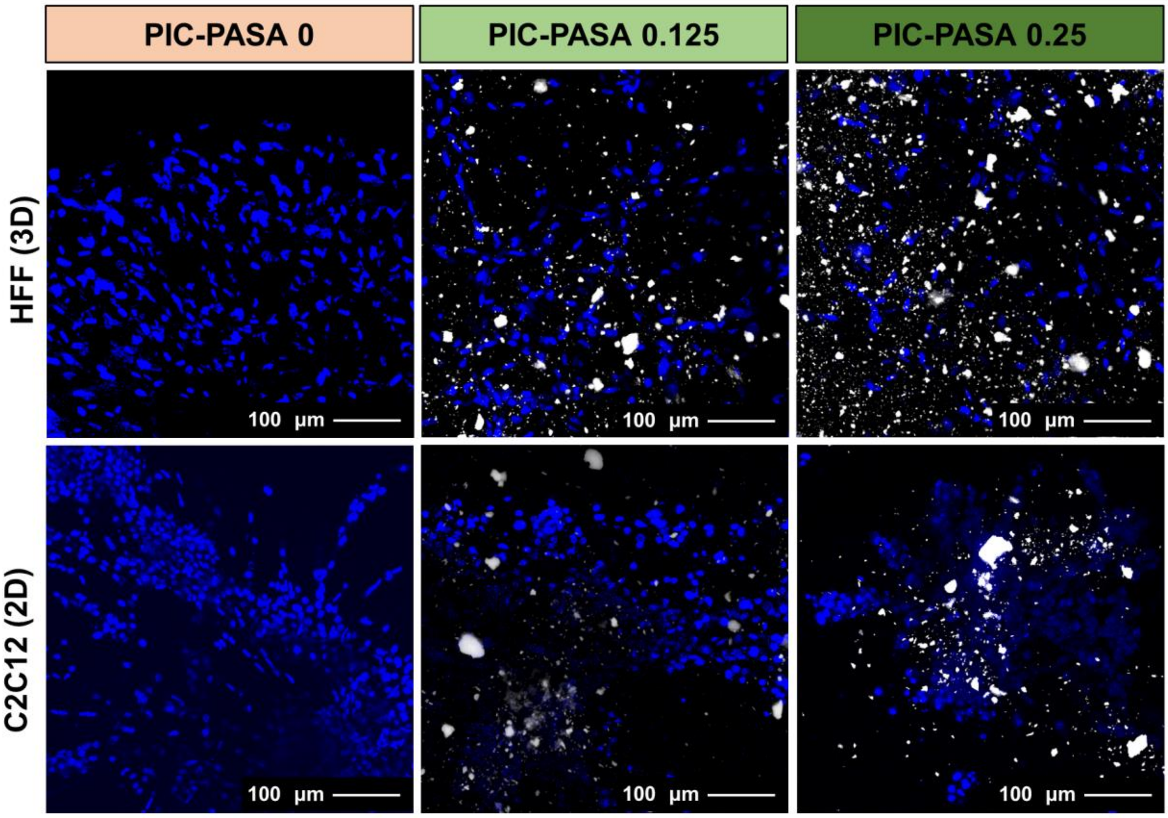

Figure S4: PASA particles in PIC-PASA hydrogels. Confocal fluorescent images of PIC-PASA 0, 0.125, and 0.25 mg/mL hydrogels seeded with HFF (upper panel) and C2C12 (lower panel) cells at T = 37 °C. Nucleus stained with DAPI (blue) and PASA particle represented as white dots (inverted form). The images were represented as maximum projection of Z-stack with thickness of 50 µm and 100 µm in case of upper and lower panel, respectively. Scale bar = 100 µm.

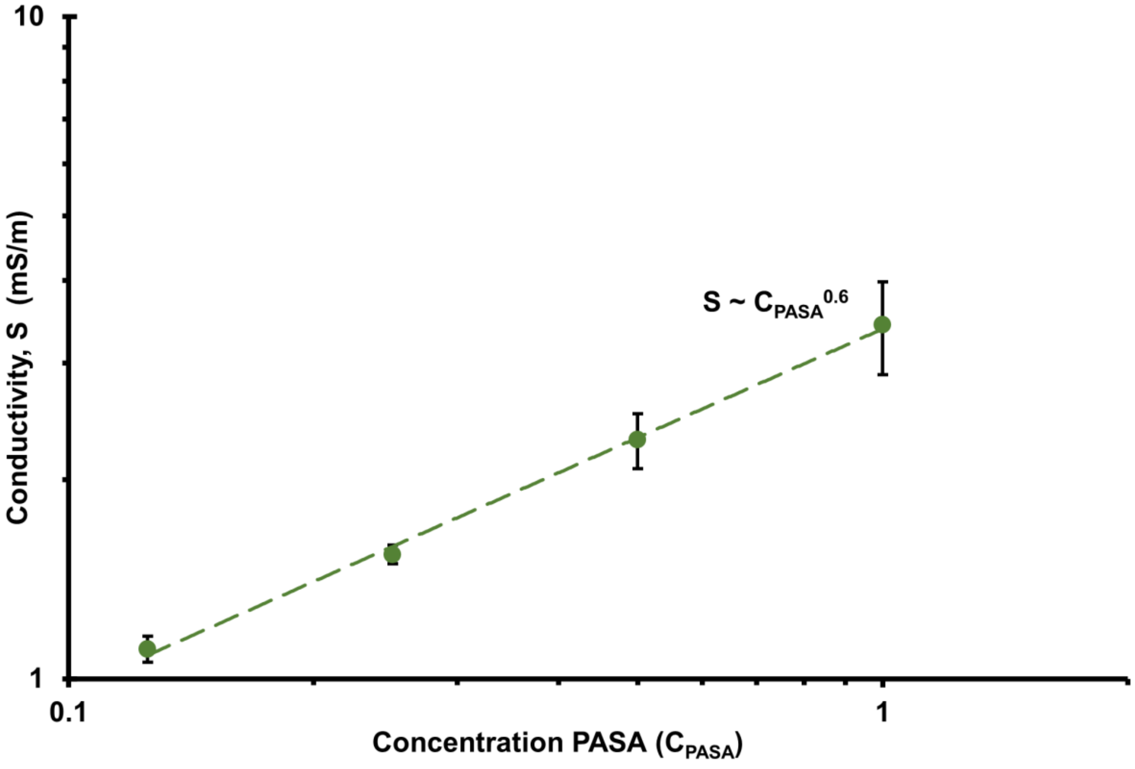

Figure S5: Conductivity of PIC-PASA hydrogels. Conductivity of PIC-PASA hydrogel as a function of PASA concentration at 37 °C; *n*=4.

Macro used to analyze fusion index

```
//Jyoti Kumari, Jelle Postma, Radboud University 2022
//based on ViaFuse-F from DOI = 10.1186/s13395-021-00284-3
//adapted to calculate nuclei number ratio of in/outside myotubules
//added a small radius pseudo-flatfield correction
//requires Biovoxxel

//setup
dir = getDir("Choose a Directory"); //user chooses what to name
title = "Untitled";
width=600; height=600;
Dialog.create("Enter File Names");
Dialog.addString("Directory:", dir);
Dialog.addString("Save Fusion Index Results As:", title); //user chooses what to name results
Dialog.show();
dir = Dialog.getString()
```

```
fileName = Dialog.getString() + " nucleiOutside VS nucleiTotal";

//assigning channel numbers to named_variables
dapi_channel = 1;
myh_channel = 2;

//begin analysis
imagename = getTitle();
width=600; height=600;
Dialog.create("Enter Multiplication Value");
Dialog.addNumber("Nuclei Multiplication Value", 1);
Dialog.addNumber("Myotube Multiplication Value", 1);
Dialog.show();
multip1 = Dialog.getNumber();
multip2 = Dialog.getNumber();
setSlice(dapi_channel);
dapi = getInfo("slice.label");
run("Multiply...", "value=&multip1 slice");
setSlice(myh_channel);
myh = "C2-" + imagename;
run("Multiply...", "value=&multip2 slice");

run("Split Channels");
selectWindow("C1-" + imagename);
dapi = getTitle();

run("Pseudo flat field correction", "blurring=50 hide");

setAutoThreshold("Default dark");
setOption("BlackBackground", true);
run("Convert to Mask");
run("Median...", "radius=1");
run("Close-");
run("Watershed");
wait(100);

selectWindow(myh);
run("Gaussian Blur...", "sigma=2");
setAutoThreshold("Default dark");
setOption("BlackBackground", true);
```

```
run("Convert to Mask");
run("Close-");
run("Median...", "radius=1");
wait(100);

//image subtraction
selectWindow(dapi);
run("Set Measurements...", "area add redirect=None decimal=0");
run("Analyze Particles...", "size=50-250 display exclude add");
nucleinumbertotal = nResults;
close("Results");

run("Select None");
roiManager("reset");
imageCalculator("Subtract create", dapi, myh); //results in nuclei outside of myotubules
run("Set Measurements...", "area add redirect=None decimal=0");
selectWindow("Result of " + dapi);
run("Analyze Particles...", "size=50-250 display exclude add"); //50 micorns square = excluded, <250
microns squred = included

//number of particles
nucleinumberoutside = nResults;

selectWindow("Results");
run("Close");
roiManager("reset");
close("ROI Manager");

print("Total number of nuclei and number of nuclei outside of myotubules");
print("image name = " + imagename);
print(nucleinumbertotal);
print(nucleinumberoutside);

File.append(nucleinumberoutside, dir + fileName + ".csv");
File.append(nucleinumbertotal, dir + fileName + ".csv");

run("Close All");

print("done!")
```

Video S1: Three-dimensional projection of PASA particles in PIC-PASA 0.125 hydrogels**.** Confocal fluorescent images of HFF cells with PASA particle (inverted form, white) and nucleus (stained with DAPI, blue), showing distribution of PASA inside PIC-PASA 0.125 hydrogel. PASA was represented as white dots and PIC as a black background. Z-project thickness = 50 μm. Scale bar = 100 μm.

Video S2: Three-dimensional projection of PASA particles in PIC-PASA 0.25 hydrogels**.** Confocal fluorescent images of HFF cells with PASA particle (inverted form, white) and nucleus (stained with DAPI, blue), showing distribution of PASA inside PIC-PASA 0.25 hydrogel. PASA was represented as white dots and PIC as a black background. Z-project thickness = 50 μm. Scale bar = 100 μm.
